# Etiologic spectrum of interstitial lung diseases in Chinese children older than 2 years of age

**DOI:** 10.1186/s13023-019-1270-7

**Published:** 2020-01-22

**Authors:** Xiaolei Tang, Huimin Li, Hui Liu, Hui Xu, Haiming Yang, Jinrong Liu, Shunying Zhao

**Affiliations:** Department of Respiratory Medicine, Beijing Children’s Hospital, National Center for Children’s Health, Capital Medical University, China, No. 56 Nailishi Road, Xicheng District, Beijing, 100045 China

**Keywords:** Childhood, Interstitial lung diseases, Diffuse lung disease, Diffuse parenchymal lung disease

## Abstract

**Background:**

Childhood interstitial lung diseases (ILD) (chILD) refer to a rare heterogeneous group of disorders. Global collaborations have been working on the etiologies and classification scheme of chILD. With the development of medical technologies, some new diseases were identified to be associated with chILD and its etiologic spectrum is expanding. The aim of this study is to describe the etiologic spectrum of chILD in children older than 2 years of age and summarize the approaches to diagnosis of chILD.

**Methods:**

We made a retrospective analysis of children older than 2 years of age with chILD who referred to Beijing Children’s Hospital from 21 provinces all over China from 2013 to 2018. After excluding pulmonary infection, congenital heart disease, bronchopulmonary dysplasia, bronchiolitis obliterans and bronchiectasis, 133 patients were included and categorized by etiology. Clinical manifestations, high-resolution computed tomography, laboratory data, genetic data and pathologic findings were all collected and reviewed.

**Results:**

Systemic disease associated ILD were the most common causes, accounting for 49.6% of the patients, followed by alveolar structure disorder-associated ILD (27%), exposure related ILD (13.5%), and disorders masquerading as ILD (3.8%). In systemic disease associated ILD, in addition to common etiologies such as vasculitis (10.5%) and connective tissue diseases (9.0%), primary immunodeficiency diseases (PID) associated ILD (9.8%), interstitial pneumonia with autoimmune features (6.8%), and metabolic diseases (6.8%) were not rarely found. Some newly reported etiologies such as STING–associated vasculopathy with onset in infancy, COPA syndrome and *STAT3* mutation were included in PID associated ILD. Genetic tests contributed to 15% of the diagnoses which mainly distributed in PID associated ILD, metabolic diseases and surfactant dysfunction disorders, and contributed to the final diagnoses more than lung biopsies (13.5%) and biopsies of rashes or other tissues (12%).

**Conclusions:**

This study first demonstrated an etiologic spectrum of chILD in Chinese children older than 2 years of age and summarized the approaches to diagnosis. The etiologic spectrum of chILD is expanding with more genetic etiologies being recognized.

## Introduction

Childhood interstitial lung diseases (chILD) refer to a rare heterogeneous group of disorders associated with significant morbidity and mortality, characterized by abnormalities of the distal lung units and disordered gas exchange [1–3]. In addition to interstitial lung tissue, other parenchymal components such as vessels, epithelium, airways or pleura are usually also involved, so it’s also called diffuse parenchymal lung disease (DPLD), or diffuse lung disease (DLD). Global collaborations have been working on the etiologies and classification scheme of chILD / DLD, while Chinese data are still lacking. One existing classification scheme of chILD was proposed by Clement A et al, dividing chILD into four groups: “exposure related ILD”, “systemic disease associated ILD”, “alveolar structure disorder-associated ILD”, “ILD specific to infancy” [4]. Another common used classification scheme was proposed by the chILD Research Co-operative of North America for DLD in children, broadly dividing DLD into “disorders more prevalent in infancy” and “disorders not specific to infancy” according to the ages younger or older than 2 years [5]. More previous studies have been working on “disorders more prevalent in infancy” in children younger than 2 years of age [6, 7], while less previous cases were reported in children older than 2 years of age [8]. Recently, some new etiologies of chILD such as the coatomer protein, subunit alpha (COPA) syndrome and stimulator of interferon genes (STING)–associated vasculopathy with onset in infancy (SAVI) were reported with the development of genetic technologies, which expanded the etiologic spectrum of chILD. The aim of this study was to describe and expand the etiologic spectrum of chILD in children older than 2 years of age, assess the diagnostic value of clinical manifestations, high-resolution computed tomography (HRCT), laboratory and other investigations, genetic tests and biopsies and summarize the approaches to diagnosis of chILD.

## Methods

### Inclusion and exclusion criteria

Based on the combined experiences of clinicians, radiologists, and pathologists in Beijing Children’s Hospital, National Center for Children’s Health, we made a retrospective analysis of children older than 2 years of age with chILD who referred from 21 provinces all over China to the Department of Respiratory Medicine (Ward 2) of Beijing Children’s Hospital from Jan 2013 to July 2018. The inclusion criteria referred to the diagnostic criteria of “chILD syndrome” proposed by American Thoracic Society [9], with at least the presence of three of the followings: (1) respiratory symptoms (cough, difficult breathing, or exercise intolerance), (2) respiratory signs (tachypnea, retractions, crackles, digital clubbing, failure to thrive, or respiratory failure), (3) hypoxemia, (4) diffuse chest infiltrates on chest radiograph or HRCT scan. ChILD caused by pulmonary infection, growth-abnormal diseases including congenital heart disease and bronchopulmonary dysplasia, and airway-related diseases including bronchiolitis obliterans (BO) and bronchiectasis (caused by cystic fibrosis, primary ciliary dyskinesia, et al) were all excluded. Different from the “chILD syndrome” exclusive criteria [9], BO was excluded in this study because that both BO and bronchiectasis are common airway-related diseases which can be easily identified from the HRCT. These airway-related diseases are a heterogeneous group of disorders with different etiologies which we think need to be discussed separately. Recurrent aspiration and primary immunodeficiency disease (PID) which may not be easily identified were not excluded in this study. Information including clinical symptoms and signs, family history, past history, environment contact history and investigations such as blood and urine routine, liver and renal function, immunologic function tests, autoantibody tests, screening of metabolic disease, chest HRCT, echocardiography, 24-H esophageal PH monitoring, bronchoalveolar lavage (BAL) cellular analysis, genetic tests, and biopsies of lungs, rashes and other tissues were all collected and evaluated.

### Etiological classification

Referring and modifying the classification schemes for chILD / DLD proposed by Clement A et al [4] and the chILD Research Co-operative of North America [5, 9], we used the following classification scheme for chILD children old than 2 years of age, which divided chILD into five categories: (1) exposure related ILD, (2) systemic disease associated ILD, (3) alveolar structure disorder-associated ILD, (4) disorders masquerading as ILD, (5) unclassified. The category “ILD specific to infancy” was not mentioned in this study because it focused on chILD in children older than 2 years of age only. Compared to former classification schemes, a new term “interstitial pneumonia with autoimmune features (IPAF)” was applied in our study with the diagnostic criteria recommended by European Respiratory Society/American Thoracic Society for patients with an ILD and clinical features that suggest an underlying autoimmune process but do not meet established criteria for a connective tissue disease (CTD) [10]. IPAF was classified into category of systemic disease associated ILD. PID associated ILD was classified into the category of systemic disease associated ILD because that ILD may be one of the systemic involvements caused by the autoimmune or auto-inflammatory features of PID. Diffuse alveolar hemorrhage (DAH) as a heterogeneous group of disorders were included and separated by etiologies, those caused by systemic diseases such as vasculitis, CTD, IPAF were classified into systemic disease associated ILD, and those with negative serology and no proof of systemic disease were classified into category of alveolar structure disorder-associated ILD and were termed “DAH with no proof of systemic disease”.

## Result

One hundred and thirty-three children older than 2 years of age with chILD were included in this study. Eighty-four children (63.2%) were male. The mean age at the time of administration was 6.0 years (ranged from 2.0–14.3 years).

### Etiologic spectrum

The classification of chILD by etiology and frequency of disease was showed in Table 1. Eighteen patients (13.5%) were assigned to the category of exposure related ILD. The most common cause in this category was hypersensitivity pneumonitis (HP) (*n* = 10), among whom, six were induced by avian antigens, three were induced by chemicals, one was induced by Aspergillus.

**Table 1:** Classification of chILD in children older than 2 years of age by etiology and frequency of different category (*N*=133)

I: Exposure related ILD N=18 (13.5%)		
Hypersensitivity pneumonitis	N=10 (7.5%)	
Drug-induced hypersensitivity reaction	N=2 (1.5%)	
Recurrent aspiration	N=6 (4.5%)	
II: Systemic disease associated ILD N=66 (49.6%)		
Connective tissue diseases	N=12 (9.0%)	
Juvenile dermatomyositis		N=7
Juvenile idiopathic arthritis		N=3
Systemic lupus erythematosus		N=2
Interstitial pneumonia with autoimmune features	N=9 (6.8%)	
Vasculitis	N=14 (10.5%)	
Primary immunodeficiency diseases associated ILD	N=13 (9.8%)	
STING–associated vasculopathy with onset in infancy		N=3
COPA syndrome		N=1
Cytotoxic T-lymphocyte associated protein-4 deficiency		N=1
*STAT3* mutation		N=1
Autoimmune lymphoproliferative syndrome		N=1
Chronic granulomatous disease		N=3
Common variable immunodeficiency disease		N=1
Inflammatory bowel disease with neutropenia		N=1
Combined immunodeficiency disease		N=1
Langerhans cell histiocytosis	N=7 (5.3%)	
Metabolic diseases	N=9 (6.8%)	
Methylmalonic acidemia and homocysteinemia		N=7
Niemann-Pick disease		N=2
Malignant infiltrates	N=2 (1.5%)	
Lymphoma		N=1
Pulmonary metastases from thyroid carcinoma		N=1
III. Alveolar structure disorder-associated ILD N=36 (27.0%)		
Surfactant dysfunction disorders	N=5 (3.8%)	
*SFTPC* mutation		N=3
*ABCA3* mutation		N=2
Diffuse alveolar hemorrhage with no proof of systemic disease	N=27 (20.3%)	
Cryptogenic organizing pneumonia	N=4 (3.0%)	
IV: Disorders masquerading as ILD N=5 (3.8%)		
Diffuse pulmonary lymphangiomatosis	N=3 (2.3%)	
Pulmonary hypertensive vasculopathy	N=2 (1.5%)	
V:Unclassified N=8 (6.0%)		
N: number		

About a half of the patients (*n* = 66, 49.6%) were classified into the category of systemic disease associated ILD. In this category, vasculitis, PID associated ILD and CTD were the most common causes. In the patients with PID associated ILD, referring classification proposed by the International Union of Immunological Societies [11], auto-inflammatory disorders (including SAVI and COPA syndrome), diseases of immune dysregulation (including cytotoxic T-lymphocyte associated protein-4 (*CTLA4*) deficiency, autosomal dominant signal transducer and activator of transcription 3 (*STAT3*) mutation and autoimmune lymphoproliferative syndrome (ALPS)) and chronic granulomatous disease (CGD) were common. In addition, one patient was diagnosed with common variable immunodeficiency disease (CVID) associated with granulomatous-lymphocytic ILD (GLILD) confirmed by lung biopsy. Nine patients were diagnosed with IPAF, with antinuclear antibodies (ANA) > =1:320 titre in five children, positive anti-cyclic citrullinated peptide (CCP) antibody in three children, and positive anti-Sjögren’ s syndrome A (SSA) antibody in one child. Other causes in this category included metabolic diseases, langerhans cell histiocytosis (LCH) and malignant infiltrates.

Thirty-six patients (27.0%) were assigned to the category of alveolar structure disorder-associated ILD. Most of them were DAH with no proof of systemic disease, followed with surfactant dysfunction disorders. Five patients (3.8%) were assigned to the category of disorders masquerading as ILD including diffuse pulmonary lymphangiomatosis (DPL) and pulmonary hypertensive vasculopathy. Eight patients (6%) were unclassified.

There were five patients who had two coexisting diseases. Two patients who had CGD coexisting with HP and one patient who had CGD coexisting with IPAF were assigned as CGD. Two patients who had surfactant protein C *(SFTPC)* mutation coexisting with IPAF was assigned as surfactant dysfunction disorder.

### Diagnostic value of clinical manifestations and investigations

#### Clinical manifestations

**Fig. 1:**
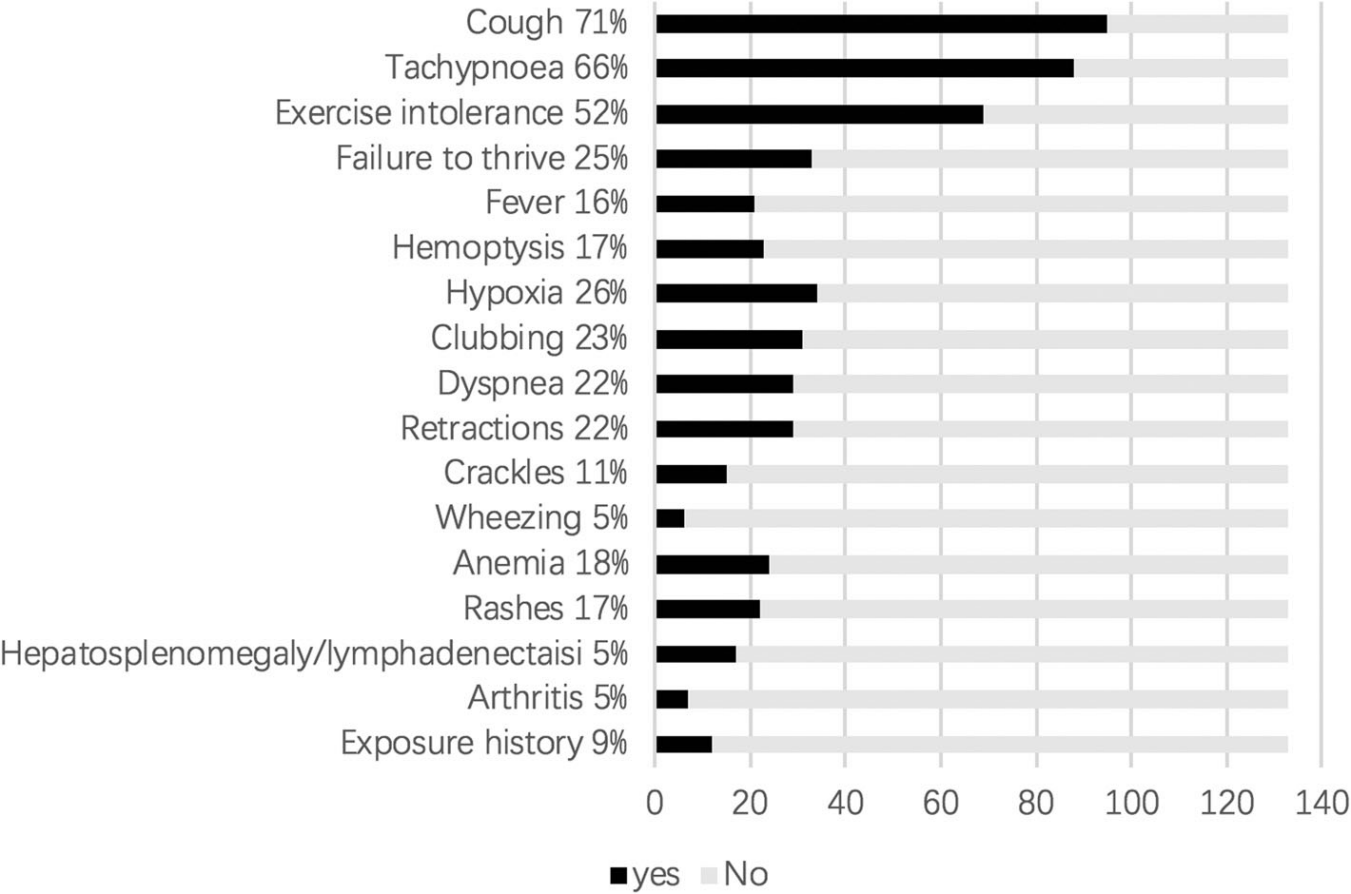
Symptoms and signs of chILD in children older than 2 years of age

The most common symptoms and signs were cough (71%), tachypnea (66%) and exercise intolerance (52%), followed with hypoxia, failure to thrive, clubbing, et al. (Fig. 1). In addition to common symptoms, hemoptysis and anemia were presented in 17 and 18% of the patients, with a main distribution in the patients of vasculitis, and DAH with no proof of systemic disease. In addition, hemoptysis was also presented in some patients with juvenile dermatomyositis (JDM), systemic lupus erythematosus (SLE), and IPAF with or without anemia. Rashes were found in 17% of the patients, with a main distribution in the patients with LCH (*n* = 6) and JDM (*n* = 7), and also in the patients with SLE, drug-induced hypersensitivity reaction, vasculitis, SAVI and CTLA4 deficiency. Myasthenia was presented in three out of the seven JDM patients. Arthritis was presented in one out of the three JIA patients before admission and developed in the later lives in the other two JIA patients during the follow-up. Arthritis was also found in some patients with SLE, JDM, vasculitis, SAVI and COPA syndrome. Hepatosplenomegaly and/or lymphadenopathy was mainly found in the patients with LCH, metabolic diseases (such as MMA and Niemann-Pick disease (NPD)), PID associated ILD (such as CVID, CTLA4 deficiency, ALPS and CGD) and malignant infiltrates.

#### HRCT

**Fig. 2:**
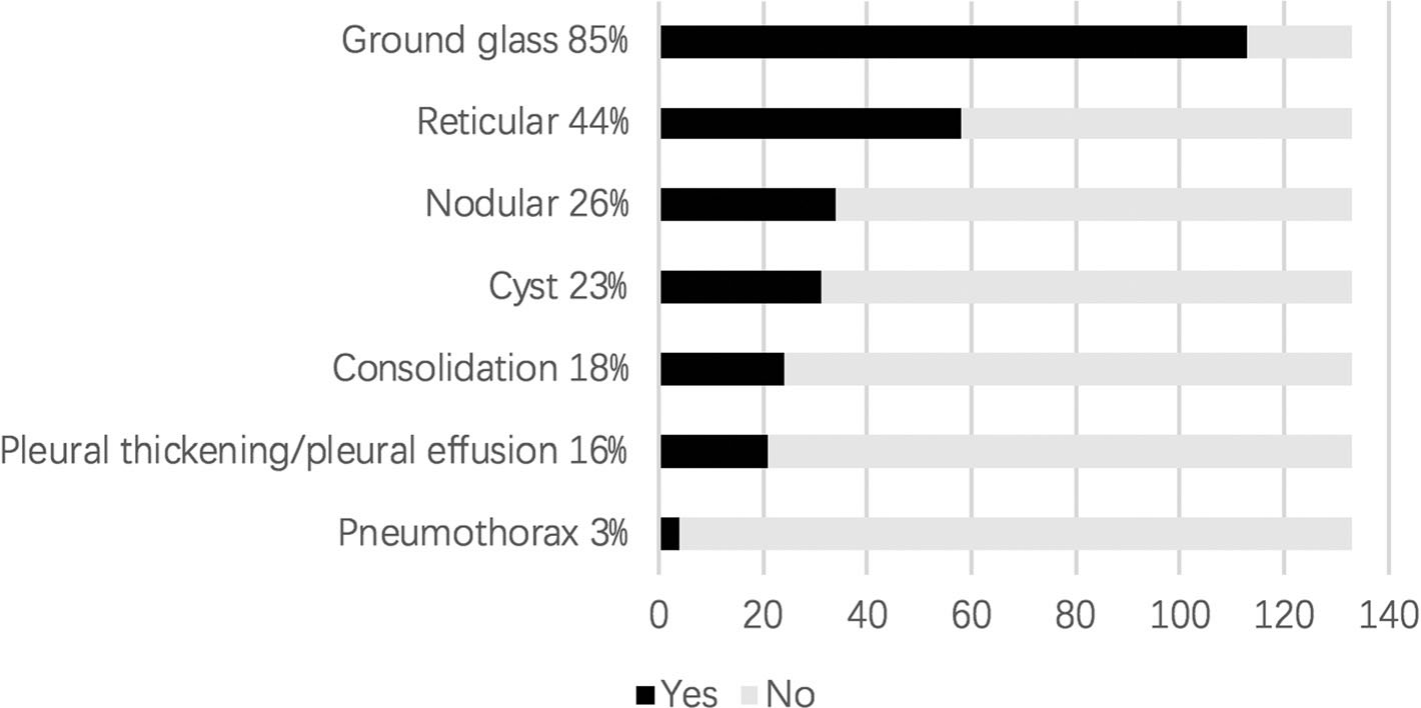
HRCT features of chILD in children older than 2 years of age

**Fig. 3:**
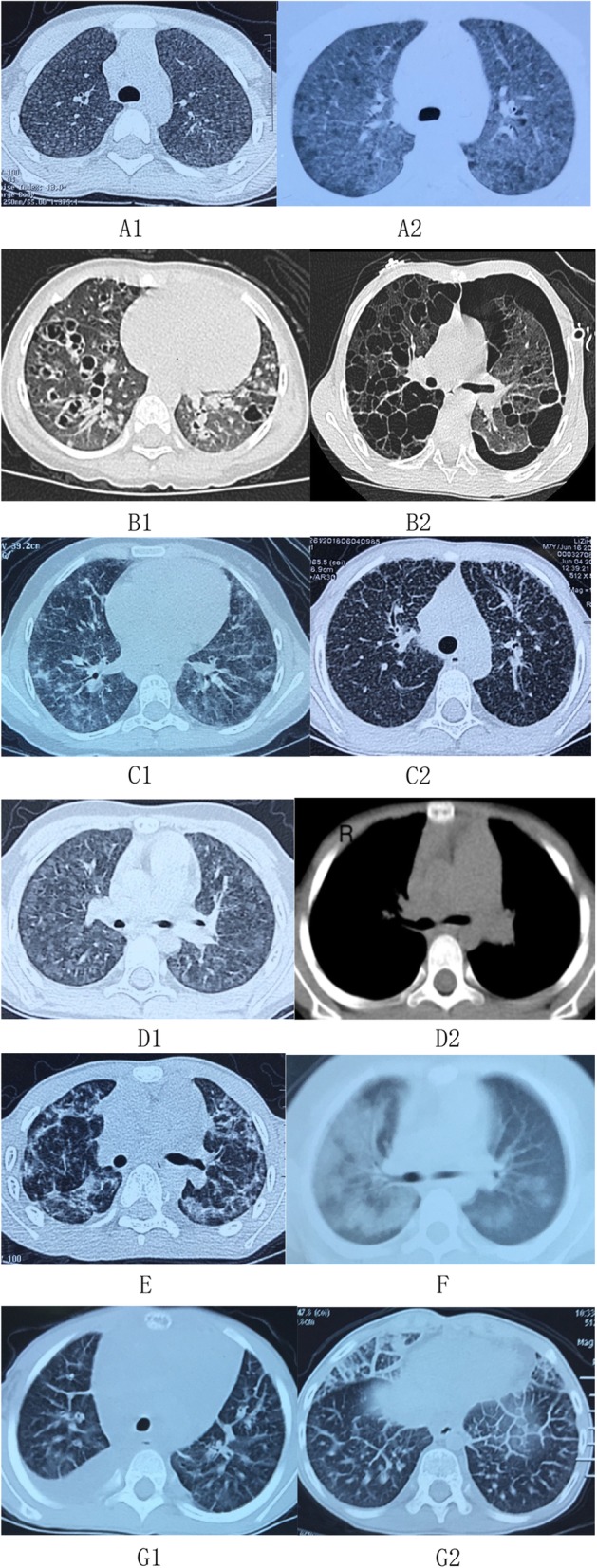
Characteristic HRCT of chILD in children older than 2 years of age. **A1** HRCT of hypersensitivity pneumonitis in a patient showing diffuse bilateral small poorly-defined centrilobular nodules. **A2** HRCT of hypersensitivity pneumonitis in another patient showing diffuse bilateral ground-glass opacities with areas of air trapping. **B1** HRCT of langerhans cell histiocytosis (LCH) in a patient showing bilateral cysts and nodules. **B2** HRCT of LCH in another patient showing bilateral cysts and pneumothorax. **C1** HRCT of common variable immunodeficiency (CVID) with granulomatous-lymphocytic interstitial lung disease (GLILD) in a patient showing reticulonodular opacities with bilateral ground-glass macronodular opacities. **C2** HRCT of COPA syndrome in a patient showing a lymphocytic intestinal pneumonia (LIP) pattern characterized by bilateral diffuse reticulonodular opacities. **D1** and **D2** HRCT of methylmalonic acidemia (MMA) and homocysteinemia in a patient showing diffuse bilateral poorly defined ground-glass nodules and enlarged pulmonary artery. **E** HRCT of cryptogenic organizing pneumonia (COP) in a patient showing bilateral liner opacities and consolidations in peripheral distribution. **F** HRCT of diffuse alveolar hemorrhage (DAH) in a patient showing bilateral diffuse bilateral ground-glass opacities. **G1** and **G2** HRCT of a patient with diffuse pulmonary lymphangiomatosis (DPL) showing diffuse bilateral grossly thickened interlobular septal and pleural effusion

The most common findings of HRCT were ground glass patches (85%), reticular patches (44%), followed with nodules, cysts, consolidation, et al. (Fig. 2). Cysts were mainly found in the LCH patients (*n* = 6) (Fig. 3B1, B2), and also in the patients with CTD, vasculitis, DAH with no proof of systemic disease, COPA syndrome, CVID, CGD, *STAT3* mutation, and surfactant dysfunction disorders. Nodules were mainly distributed in the patients with MMA (*n* = 6) (Fig. 3D1), HP (*n* = 6) (Fig. 3A1), and LCH (*n* = 4) (Fig. 3B1). Pleural thickening/pleural effusion were found most common in CTD and DPL (Fig. 3G1). Grossly thickened interlobular septal was a characteristic sign found in all the three patients with DPL (Fig. 3G1). Pneumothorax was found in the patients with LCH (*n* = 3) (Fig. 3B2) and JDM (*n* = 1). Characteristic HRCT features which may lead to a diagnosis were mainly found in the patients with HP, LCH, PID, MMA, COP, DAH and DPL (showed in Fig. 3).

#### Laboratory and other investigations

Autoantibody tests including antinuclear antibodies (ANA), double-stranded deoxyribonucleic acid (ds-DNA), extractable nuclear antigen (ENA), anti-neutrophil cytoplasmic antibodies (ANCA), rheumatoid factors (RF) and anti-cyclic citrullinated peptide (CCP) antibody were performed in 96%, 96%, 93%, 92%, 79% and 68% of the patients and were found to be positive not only in all the patients with CTD, vasculitis, and IPAF, and also in some patients with PID associated ILD such as COPA syndrome, SAVI, and CGD coexisting with IPAF and some patients with SFTPC genetic mutation coexisting with IPAF. Myositis-specific autoantibodies such as anti-MDA5 antibody were positive in three out of the seven JDM patients. Immunologic function tests including serum immunoglobulin and lymphocyte subsets were performed in 95% of the patients and contributed to the diagnoses of PID (such as CVID, combined immunodeficiency disease, and *STAT3* mutation). Neutrophil respiratory burst assay was positive in all the three CGD patients. Screenings for metabolic diseases including serum homocysteine test, investigations of metabolites with tandem mass spectrometry and organic acid analysis with gas chromatography were performed in 30%, 26% and 26% of the patients and mainly contributed to the diagnoses of MMA. Niemann-pick cells were found in the two patients with NPD through bone marrow aspiration. Bronchoscopy with BAL cellular analysis was performed in 60% of the patients. A large number of hemosiderin-laden macrophages in BAL were found in 32% of the patients (*n* = 43), which mainly distributed in the patients with DAH with no proof of systemic disease and vasculitis, and were also found in some patients with JDM, JIA, SLE, IPAF, and SAVI. Among those patients, eight patients (19%) had no symptoms of hemoptysis or anemia. Echocardiography was performed in 98.3% of the patients. Pulmonary arterial hypertension (PAH) was found in 12% of the patients with a main distribution in MMA (*n* = 6), and pulmonary hypertensive vasculopathy (*n* = 2). Pericardial effusion was found in the two patients with DPL. 24-H esophageal PH monitoring was performed in 21 patients (24%). Gastroesophageal reflux (GER) was found in 14 (10.5%) patients, six of whom were diagnosed with chILD caused by recurrent aspiration.

#### Genetic tests

Genetic tests were performed in 53 patients (40%), with the technique of next generation sequencing and Sanger sequencing. Among them, 20 patients (38%) had positive results. Genetic tests contributed to 15% of the final diagnoses, including *SPTPC* mutation (*n* = 3), *ABCA3* mutation (*n* = 2), SAVI (*TMEM173* mutation) (*n* = 3), COPA syndrome (*COPA* mutation) (*n* = 1), CGD (*CYBB* mutation) (*n* = 3), CTLA4 deficiency (*CTLA4* mutation) (*n* = 1), *STAT3* mutation (*n* = 1), MMA (*MMACHC* mutation) (*n* = 5), NPD type C1 (*NPC1* mutation) (*n* = 1) and NPD type B (*SMPD1* mutation) (*n* = 1). The disease-causing genes needed further exploration in one patient with inflammatory bowel disease with neutropenia and one patient with combined immunodeficiency disease.

#### Biopsies of lung or other tissues

Lung biopsy data was available in 35% of the patients (*n* = 47), among whom, 43% of the patients underwent video assisted thoracoscopic biopsies and 57% of the patients underwent transbronchial lung biopsies. Lung biopsies contributed to the final diagnoses in 13.5% of the patients, mainly distributing in the patients with HP, PID associated ILD, malignant infiltrates and COP. Skin biopsies were performed in 12 patients, among whom, nine patients had positive pathologic findings including LCH (*n* = 6), SAVI (*n* = 1) and vasculitis (*n* = 2). Muscle biopsies were performed in four out of the seven patients with JDM and all of them had characteristic findings of myositis. Renal biopsies were performed in one MMA patient and one SLE patient with positive results. Thyroid biopsy was performed in the thyroid carcinoma patient with positive result. Biopsy of rashes and other tissue totally contributed to about 12% of the diagnoses.

## Discussion

This study is the first large sample analysis of the etiologic spectrum of chILD in Chinese children older than 2 years of age. The “chILD” in our study was narrowly defined and was specific to the non-growth abnormal, non-airway related and non-infectious chILD, which were difficult to be diagnosed. Though it’s a single center study, the patients were referred from all over of mainland China, so it had a good representation of chILD in China.

We found a higher percentage of patients with systemic disease associated ILD in our cohort than in other cohorts with the same age range [7, 8]. The systemic disease associated ILD was expanding mainly in the following aspects: (1) Though having excluded infectious disease, PID associated ILD including some newly reported diseases accounted for a higher proportion of chILD than expected, which mainly distributed in auto-inflammatory disorders, diseases of immune dysregulation and CGD. (2) A new term “IPAF” was first added into category of systemic disease associated ILD for children [10]. (3) ILD associated with MMA and homocysteinemia, which were rarely mentioned before, were not rarely found in our cohort. This result, on the one hand, may be because of genetic-tests performed in nearly 40% of the patients in our cohort, so as to improve the diagnoses of PID and metabolic disease. On the other hand, a systemic screening of autoantibodies in our cohort may improve the diagnostic rate of CTD, IPAF and vasculitis. In addition, the chILD in our cohort was narrowly defined and the different exclusive criteria applied between our study and other studies may also result in the differences.

As for PID associated ILD, it has been recognized that PID may be associated with immune-mediated ILD especially in auto-inflammatory disorders, diseases of immune dysregulation, and predominantly antibody deficiencies such as CVID [11–20]. Two new auto-inflammatory disorders SAVI and COPA syndrome which has been reported to be associated with ILD [15–17] were both found in our study cohort. In addition, diseases of immune dysregulation such as CTLA4 deficiency, *STAT3* mutation and ALPS were also found in this study cohort. CGD has been reported to be associated with ILD which mainly caused by coexisting HP [21, 22]. Two out to the three CGD patient in this study were associated with HP and one of them has been reported by our colleagues previously [23]. The third CGD patient in our cohort was coexisted with IPAF, which indicated the possibility that CGD associated ILD may be immune-mediated. In PID associated ILD, a pathological and HRCT pattern characteristic of lymphocytic intestinal pneumonia (LIP), follicular bronchiolitis (FB), pulmonary nodular lymphoid hyperplasia, and reactive lymphoid infiltrates has been reported and termed “granulomatous-lymphocytic interstitial lung disease (GLILD)” [12, 24–26]. GLILD was mainly reported in CVID and has also been reported in other PID such as CTLA4 deficiency, ALPS, lipopolysaccharide responsive beige-like anchor protein (LRBA) deficiency, et al. [13, 15, 20]. In our study cohort, GLILD was found in one CVID patient with characteristic pathological and HRCT findings. In addition, it was also found in one patient with ALPS and one patient with COPA syndrome whose lung biopsies and HRCT revealing LIP pattern. Those results indicate that a radio-pathological pattern of GLILD may lead to a suspicion of PID associated ILD.

Our study first introduced the term “IPAF” to the etiology spectrum of ILD in children, which has been applied to adult ILD since 2015 [10]. Patients who meet the diagnostic criteria of IPAF have features that suggest underlying autoimmune process but do not meet established criteria for a CTD. These patients may develop a CTD or vasculitis in later life and need to be followed up. There were two patients who were initially diagnosed with IPAF who developed arthritis in their later lives during follow-up, which made the final diagnoses changed to be JIA. Moreover, CTD, vasculitis and IPAF may be just a part of manifestations of PID which may present with autoimmunity and auto-inflammation. In this cohort, one COPA patient presented with the manifestations of JIA, and two SAVI patient presented with the manifestations of ANCA-associated vasculitis. Therefore, we suggest that genetic tests should be considered for the patients with autoimmune diseases associated with ILD, especially in those who presented with ILD as the initial or main manifestation.

Among the seven patients who were diagnosed with MMA and homocysteinemia, homozygotic or compound heterozygous *MMACHC* mutations were found in four of them, which suggested type CblC. MMA type CblC has been previously reported to be associated PAH, while ILD was rarely reported previously. Our colleagues first reported four MMA patients who were associated with ILD previously [27] and two of them were included in this cohort. A characteristic HRCT pattern of diffuse poorly defined centrilobular nodules coexisting with PAH were found in five out of the seven patients, which may be suggestive for the diagnosis. Serum homocysteine test is a quick method for screening of MMA, and we suggest it to be performed in all the patients with ILD coexisted with PAH and in the patients with ILD of known causes.

In exposure related ILD, ILD caused by recurrent aspiration in older children may easily missed diagnosed. Most children who were diagnosed with recurrent aspiration in this cohort were asymptomatic or with mild symptoms. Our colleagues previously reported two patients including in this cohort who had histological pattern of bronchiolitis obliterans organizing pneumonia (BOOP) due to recurrent aspiration caused by GER lately [28]. Similarly, GER or aspiration was reported as an uncommon cause of BOOP in adults [29, 30], therefore, a 24-H esophageal PH monitoring, upper gastrointestinal imaging (UGI), and laryngoscope may be considered for the patients with an ILD of unknown cause no matter with gastrointestinal symptoms or not. On the other hand, GER may be a coexisting disease in some ILD with other causes, so excluding the other causes are also needed.

**Fig. 4:**
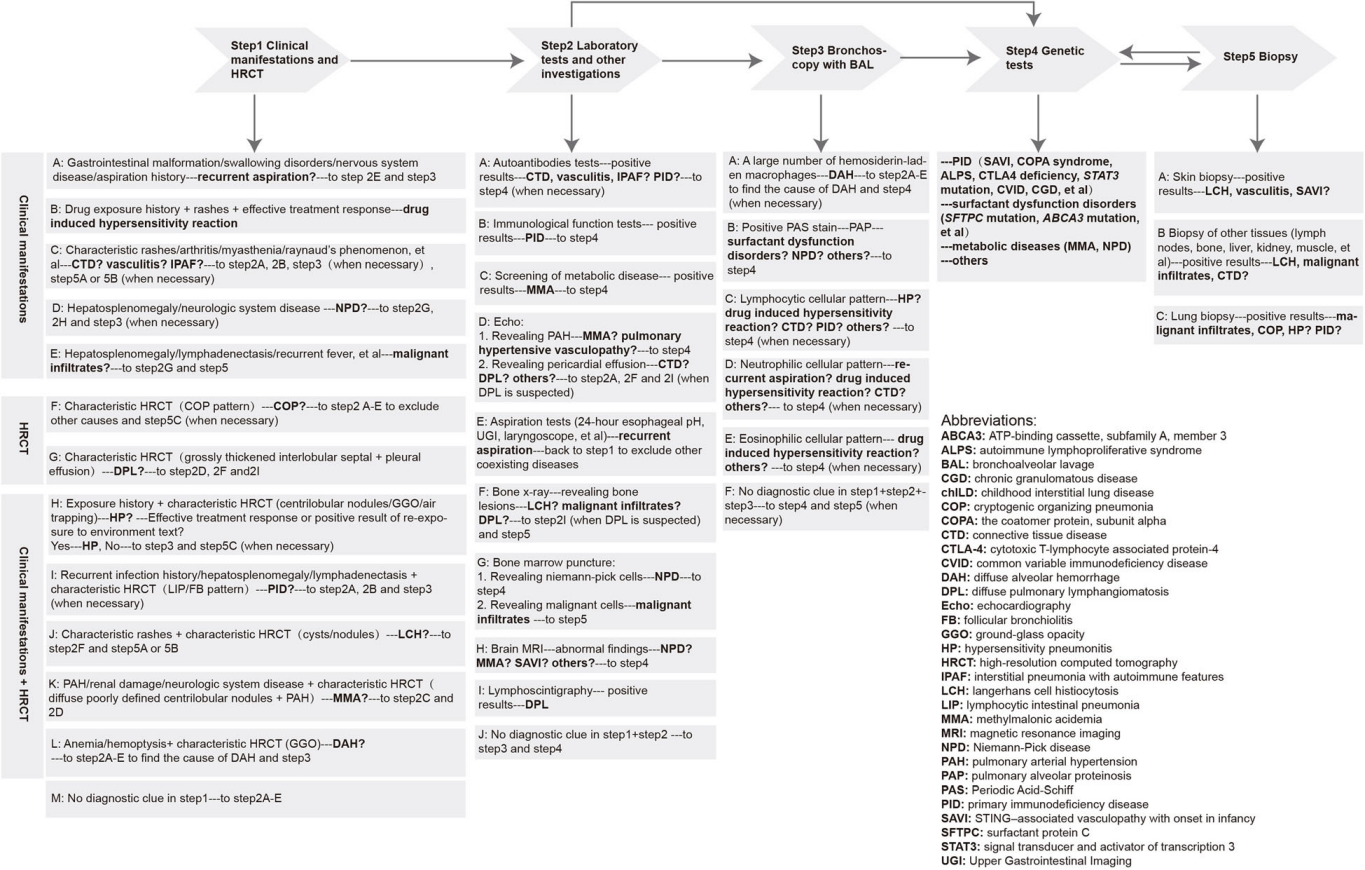
Diagnostic algorithm of chILD in children older than 2 years of age

Regarding the results of our study, a diagnosis algorithm of chILD in children older than 2 years of age was summarized in Fig. 4. The approaches to diagnosis was mainly based on five steps: (1) clinical manifestations with chest HRCT, (2) laboratory tests and other investigations, (3) bronchoscopy with BAL, (4) genetic tests and (5) biopsies. As for invasive tests, bronchoscopy with BAL cellular analysis is a relatively safe procedure. It can be diagnostic in diseases such as pulmonary alveolar proteinosis (PAP) and DAH. The recognition of a predominantly inflammatory cellular pattern in the BAL may narrow the differential diagnosis of other ILD, even though such patterns are nonspecific [9, 31, 32]. A European Respiratory Society (ERS) task force recommend that BAL should be performed in every child presenting with ILD [31]. Another guideline of ATS recommend that the degree of uncertainty about the type of ILD, the likelihood that the BAL will provide helpful information, the patient’s cardiopulmonary stability, the presence or absence of a bleeding diathesis, and the patient’s values and preferences should be considered about whether to perform a BAL [32]. In our study, bronchoscopy with BAL cellular analysis was performed in 60% of the patients and mainly contributed to the diagnoses of DAH (especially in those with no symptoms of hemoptysis or anemia) and exclusion of infection. As for lung biopsy, it is widely accepted that the potential benefits of lung biopsy outweigh the risks in most children with acute respiratory deterioration, prolonged lung disease, or unresolved lung disease [9, 33–35]. In our study, lung biopsy mainly contributed to the final diagnoses of malignant infiltrates, COP and HP. In the patients with PID, a GLILD pattern or LIP/FB pattern of lung biopsy could be suggestive to the diagnosis of PID, but the lung biopsies were not necessary before genetic tests unless the disease deteriorated fast and there were not sufficient time waiting for genetic tests or there was suspected coexisting infection with ineffective treatment. Lung biopsies were obviated in the diagnosis of LCH in our cohort mainly due to a minimally invasive procedure of skin biopsy which leaded to the diagnoses directly. Genetic tests, which were non-invasive, could obviate the need for lung biopsies especially for PID associated ILD, metabolic diseases and surfactant dysfunction disorders. Actually, genetic tests contributed to the final diagnoses more than lung biopsies in this cohort, therefore, we suggest to performing genetic tests before lung biopsies.

There are some limitations in our study. This is a single center study and the objects were children with chILD referred to our department of respiratory medicine. So those who already had a certain diagnosis of a systemic disease such as CTD and LCH before the onset of ILD and developed an ILD in later lives may not be referred to the department of respiratory medicine and were not included this cohort. We believe that adding those patients will make the systemic disease associated ILD account for a larger proportion in the etiology spectrum. However, from the perspective of pediatric pulmonologist, an etiology spectrum focused on the no pre-diagnosed chILD may be more practical.

## Conclusion

This study first demonstrates an etiologic spectrum of chILD in Chinese children older than 2 years of age and summarized the approaches to diagnosis. The etiologic spectrum of chILD is expanding with more genetic etiologies being recognized.

## Data Availability

All data generated or analysed during this study are included in this published article.

## Abbreviations

ABCA3: ATP-binding cassette, subfamily A, member 3
ALPS: Autoimmune lymphoproliferative syndrome
ANA: Antinuclear antibodies
ANCA: Anti-neutrophil cytoplasmic antibodies
BAL: Bronchoalveolar lavage
BO: Bronchiolitis obliterans
BOOP: Bronchiolitis obliterans organizing pneumonia
CCP: Cyclic citrullinated peptide
CGD: Chronic granulomatous disease
chILD: Childhood interstitial lung diseases
COP: Cryptogenic organizing pneumonia
COPA: The coatomer protein, subunit alpha
CTD: Connective tissue disease
CTLA-4: Cytotoxic T-lymphocyte associated protein-4
CVID: Common variable immunodeficiency disease
DAH: Diffuse alveolar hemorrhage
DLD: Diffuse lung disease
DPL: Diffuse pulmonary lymphangiomatosis
DPLD: Diffuse parenchymal lung disease
Ds-DAN: Double-stranded deoxyribonucleic acid
Echo: Echocardiography
ENA: Extractable nuclear antigen
FB: Follicular bronchiolitis
GER: Gastroesophageal reflux
GGO: Ground-glass opacity
GLILD: Granulomatous-lymphocytic interstitial lung disease
HP: Hypersensitivity pneumonitis
HRCT: High-resolution computed tomography
ILD: Interstitial lung diseases
IPAF: Interstitial pneumonia with autoimmune features
JDM: Juvenile dermatomyositis
LCH: Langerhans cell histiocytosis
LIP: Lymphocytic intestinal pneumonia
LRBA: Lipopolysaccharide responsive beige-like anchor protein
MDA5: Melanoma differentiation-associated protein 5
MMA: Methylmalonic acidemia
MRI: Magnetic resonance imaging
NPD: Niemann-Pick disease
PAH: Pulmonary arterial hypertension
PID: Primary immunodeficiency disease
RF: Rheumatoid factors
SAVI: STING–associated vasculopathy with onset in infancy
SFTPC: Surfactant protein C
SLE: Systemic lupus erythematosus
SSA: Sjögren’s syndrome A
STAT3: Signal transducer and activator of transcription 3
STING: Stimulator of interferon genes
UGI: Upper gastrointestinal imaging
